# Microbes Colonize a Baby’s Gut with Distinction

**DOI:** 10.1371/journal.pbio.0050191

**Published:** 2007-06-26

**Authors:** Liza Gross

If you’re going through an identity crisis, you might not want to consider the fact that, at the cellular level, you’re really more microbe than human. A hundred trillion single-celled organisms, representing some 400 bacterial species, inhabit the adult human gut, out-populating the cells in your body by a factor of ten. Nonetheless, it might comfort you to know that in exchange for room and board, your microbial cohorts offer several essential services, from pathogen protection to nutrient metabolism, and likely others yet to be discovered.

The colonization of the sterile newborn gastrointestinal tract by a thriving microbial community is a pivotal milestone in human development. Yet, many aspects of this process remain obscure. Where do the microbes come from? What affects how the colony changes over time? These are just a few of the questions that Chana Palmer, Patrick Brown, and colleagues shed light on in a new study. To their surprise, the researchers found that healthy babies had remarkably different microbial communities living in their guts during their first months of life.

With the help of 13 mothers, Palmer et al. collected stool samples from 14 healthy babies (one mother had fraternal twins), starting with daily samples just after birth, and tapering off over the first year. From these specimens—the next best thing to sampling the colonic space directly—the researchers profiled the microbial communities present during colonization. To determine the likely provenance of the microbes, they also collected stool samples from many of the parents and siblings, as well as vaginal and breast-milk samples from the mothers.

The researchers extracted genetic material from the samples, and used DNA microarrays and gene sequencing to reconstruct the colonization process. DNA microarrays are usually used to examine the expression levels of thousands of different genes in a cell at once, but Palmer et al. designed a custom DNA microarray that used a single gene capable of recognizing thousands of distinct microbial species. Acting as a species fingerprint, the small subunit ribosomal DNA (SSU rDNA) gene is found in every species and evolves relatively slowly, making it a favorite tool for classifying microbes, based on variations in this gene’s sequence across organisms.

To assess the performance of this novel tool, the researchers created a pool of SSU rDNA sequences from 12 diverse samples and generated community profiles of each sample using both the sequencing data (the “gold standard” approach) and their microarray. The two methods produced remarkably similar results, demonstrating that the microarray could reliably be used to classify microbes and estimate their relative abundance. Notably, nearly all of the 4,000-plus sequences analyzed were dominated by just three major taxonomic divisions.

Estimates of bacterial density during the first week of life (derived by estimating the total number of rRNA copies per gram of stool) revealed no significant link between mode of delivery and onset of colonization. Interestingly, however, the only two babies delivered by cesarean section, before the amniotic membranes had ruptured—and thus without exposure to the microbial flora present in their mother’s birth canal—had lower bacterial counts than the others for that first week.

The researchers went on to analyze all of the baby stool and familial samples with their microarray. Again, just three major taxonomic divisions predominated, yet the specific members of those large taxonomic groups, the timing of their appearance, and the duration of their stay varied widely among the babies. The fraternal twins were a striking exception—their microbial flora was by far more similar to each other than to other babies. Despite these individual variations, the profiles for each child showed a surprising degree of continuity—each baby could be recognized by its distinctive microbial flora for weeks and months at a time. By the time the babies were one year old, however, their profiles had converged toward a characteristic “adult-like microbiota,” which, the researchers point out, still retained a stamp of individuality.

The idiosyncratic nature of the early stages of colonization suggests that a baby’s initial bacterial profile largely results from incidental microbial encounters. The fact that some of the early stool samples matched their mother’s breast milk or vaginal sample supports this interpretation. Shared environment may also explain the coincidental appearance of microbes in the twins. The researchers explain the tendency of these communities to eventually converge by hypothesizing that the human–microbe symbiosis has likely evolved under strong selection and that certain well-adapted microbes repeatedly “win” the battle over the opportunistic early colonizers.

By comparing the surprising range of microbial profiles found in these healthy babies to the microbiota of infants born prematurely or with health problems, future studies can explore how diet, delivery method, or other factors might spell the difference between health and disease. The researchers’ custom-designed microarray provides a valuable resource for exploring such questions, as well as the environmental and genetic factors that shape and personalize the amazing “alien” ecosystem that lives within us.

